# Incidence and risk factors for first and recurrent ICD shock therapy in patients with an implantable cardioverter defibrillator

**DOI:** 10.1007/s10840-024-01873-0

**Published:** 2024-08-21

**Authors:** Diana My Frodi, Søren Zöga Diederichsen, Lucas Yixi Xing, Daniel Camillo Spona, Peter Karl Jacobsen, Niels Risum, Jesper Hastrup Svendsen

**Affiliations:** 1 Department of Cardiology, The Heart Center, Copenhagen University Hospital – Rigshospitalet, Inge Lehmanns Vej 7, DK-2100 Copenhagen, Denmark; 2 Department of Clinical Medicine, Faculty of Health and Medical Sciences, University of Copenhagen, Copenhagen, Denmark

**Keywords:** Implantable cardioverter defibrillator, Risk factors, ICD-therapy, Ventricular arrhythmia, Recurrent shock

## Abstract

**Background:**

Advances in medical treatment and outcomes in implantable cardioverter-defibrillator (ICD) recipients incentivize a need for improved candidate selection and identification of risk factors for ICD therapy. We examined contemporary rates of and risk factors for ICD therapy.

**Methods:**

Patients with ICD for primary (PP) or secondary prevention (SP), implanted between January 2010 and December 2020, were followed for appropriate and inappropriate incident and recurrent shock.

**Results:**

Overall, 2998 patients (mean age 61.8 ± 12.7 years, 20% female, 73% ICD carriers, and 47.1% SP) were analyzed with a median follow-up of 4.3 (interquartile range (IQR) 2.1–7.4) years. A total of 426/2998 (14.2%) patients had shock; 364/2998 (12.1%) had appropriate and 82/2998 (2.7%) inappropriate shock, with annualized event rates of 2.34 (2.11–2.59) and 0.49 (0.39–0.61) per 100 person-years, respectively. Of those with shock, 133/364 (36.5%) experienced recurrent appropriate shock and 8/364 (2.2%) received recurrent inappropriate shock, with event rates of 10.57 (8.85–12.53) and 0.46 (0.20–0.92), respectively.

In multivariable analyses, female sex was associated with a reduced risk of incident appropriate shock (hazard ratio 0.69 [95% confidence interval 0.52; 0.91]). Of other variables, only revascularization status was associated with recurrent appropriate shock in PP, and CRT-D with recurrent appropriate shock in the overall cohort.

**Conclusion:**

One in eight ICD recipients received appropriate shock 2–7 years after guideline-directed implantation. More than one-third of patients with a first shock experienced recurrent shock. Few clinical variables showed potential in predicting shocks, illustrating a need for more advanced tools to select candidates for implantation.

**Graphical Abstract:**

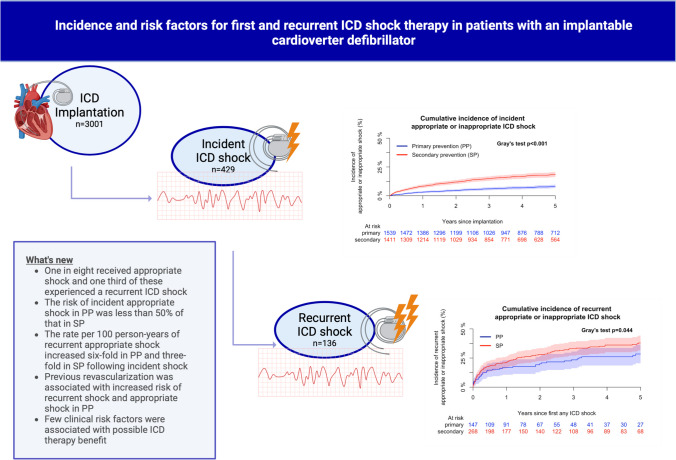

**Supplementary Information:**

The online version contains supplementary material available at 10.1007/s10840-024-01873-0.

## Introduction

Implantable cardioverter-defibrillators (ICD) alone or with cardiac resynchronization therapy (CRT-D) are widely used to prevent sudden cardiac death (SCD) in patients with an elevated risk of life-threatening ventricular arrhythmia (VA) [1, 2]. Improvements in medical treatment [3] and device effectiveness including non-shock interventions [4] have likely resulted in the decreasing incidence of SCD observed in this patient population [5]. Still, the risk of ICD shock constitutes a significant physical and psychological burden to ICD carriers. Appropriate therapy occurs in about 1 in 3 of ICD carriers [6] and approximately half of patients with incident shock will receive a recurrent shock in the following years [7]. Health deterioration that demands ICD therapy is associated with increased mortality [8] and worsened quality of life [9] despite proper detection and treatment of arrhythmia being lifesaving.

Together, the decreasing incidence of SCD and the pervasive risk of appropriate and inappropriate shocks amplify the need for improved candidate selection and measures to decrease inappropriate shocks. Although risk factors for ICD therapy have been identified [10–14], they may not apply to contemporary patients and also, the definition of appropriate ICD therapy is inconsistently defined across studies, i.e., grouping ICD shock and anti-tachycardia pacing (ATP) together [10], or combining all shocks into one group, without separation of appropriate and inappropriate shocks in subsequent analyses [7], overall affecting result comparability. Lastly, there are only sparse data on risk factors for recurrent therapy. Therefore, this study sought to evaluate the incidence of and risk factors for incident and recurrent appropriate and inappropriate ICD shock therapy in a large contemporary cohort.

## Methods

### Study design

This was a single-center study using the nationwide Danish Pacemaker and ICD Registry as data source. This registry includes prospectively collected data on all pacemaker and ICD implantations and follow-up contacts in Denmark. Upon device implantation, it is the treating electrophysiologist or device technician that enters procedural information into this registry. Follow-up data are either entered by the treating electrophysiologist or specialized device technicians during remote transmission or outpatient clinic visits. In Denmark, indications for ICD implantation follow contemporary guidelines from the European Society of Cardiology [1, 15]. All first-time recipients of a primary (PP) or secondary prevention (SP) ICD/CRT-D aged 18 years or older and with their device implanted at Copenhagen University Hospital-Rigshospitalet in the period from January 2010 to December 2020 were identified and included in the study. Baseline data were obtained on the date of implantation. For corrective measures performed after incident shock, the electronic health record at Copenhagen University Hospital-Rigshospitalet was used. Due to the implementation of a new electronic health record system in 2017, this type of data was only available in a subpopulation that received their first shock after January 1, 2017.

The patients were followed up until outcomes of interest, death, emigration, device explant, or June 2021; whichever came first.

### Outcomes

The primary outcome was incident shock combined, i.e., patients’ first appropriate or first inappropriate ICD shock, and secondary outcomes were (1) first appropriate shock and (2) first inappropriate shock separately (uncensored if both (1) and (2) were present). In patients with incident ICD shock, the recurrent study outcomes were also examined. A blanking window of 24 h was used to define separate recurrent arrhythmic events.

Appropriate shock was defined as correctly given shock therapy for ventricular tachycardia (VT) or ventricular fibrillation (VF). Inappropriate shock was defined as incorrectly received shock for supraventricular tachyarrhythmia, oversensing, or noise, faultily detected as VT or VF. Lastly, potential harm from the ICD was examined by looking at inappropriate shock, inappropriate shock and serious device-related complications (generator and lead derived), or inappropriate shock and any device-related complications. Serious generator-derived complications were systemic infection/endocarditis, local pocket infection/skin erosion, and failure with sensing/pacing. Serious lead-derived complications were other than systemic infection/endocarditis and local pocket infection/skin erosion, also lead displacement.

### Statistical analysis

Continuous variables are presented as mean ± standard deviation and were compared by *t*-test for normally distributed variables and as median (interquartile range (IQR)) and were compared by Wilcoxon rank-sum test for non-normally distributed variables. Categorical variables are presented as frequency with percentage and were compared by chi-squared test.

The 5-year cumulative incidence of study outcomes was determined using the Aalen-Johansen estimator with death as a competing risk, while the crude event rates were estimated with Poisson distribution and are presented as events per 100 person-years. Groupwise comparisons of absolute risks were conducted by Gray’s test to account for censoring and competing events.

The absolute risk reduction (ARR) for the first appropriate shock as the efficacy outcome and inappropriate shock or device-related complications as the safety outcome were used to calculate the number needed to treat (NNT) and harm (NNH). As this study did not contain any controls that were not treated with an ICD, the NNT and NNH analyses were performed under the assumption that all patients who received appropriate shock had not received this had they not been implanted with an ICD. Therefore, an absolute risk of 0% was used in the comparator group, and the absolute risk differences were calculated as the cumulative incidence (%) (i.e., cumulative incidence %-0%) and thus an NNT or NNH of 100/cumulative incidence%. NNT/NNH analyses were reported for the total population, in PP versus SP, and because of the changes in ICD programming guidelines [16, 17], between those implanted before and after the year of 2015.

Additionally, multivariable Cox proportional hazard regression analysis was performed, incorporating baseline age (per 10 year increment), sex (reference group male), Body Mass Index (BMI; underweight (BMI < 18.5 kg/m^2^), overweight (BMI 25–29.9), obesity (BMI ≥ 30.0) versus the reference group with normal weight (BMI 18.5–24.9) [18]), left ventricular ejection fraction (LVEF; ≤ 35% versus the reference group with > 35%), type of device (CRT-D versus the reference group with ICD), and previous revascularization with either Percutaneous Coronary Intervention (PCI) or Coronary Artery Bypass Graft (CABG) with the reference group being no revascularization in the final model.

A test for interaction was conducted to assess whether the impact of baseline variables on outcomes was different across PP and SP using the multivariable Cox model mentioned above. Hazard ratios (HR) are presented with 95% confidence intervals (CI). The proportional hazards assumption was examined by the scaled Schoenfeld residuals test. The study outcomes were investigated in the entire cohort as well as in PP and SP groups, separately. Patients who experienced their first ICD shock were also analyzed for recurrent events, with the date of the first event set as the index. Here, the age at index was employed in the multivariable Cox models. To better understand ICD benefit, it was also noted if patients with or without shock, both appropriate and inappropriate, also experienced appropriate ATP and/or device-related complications. An important contributing factor to occurrence of shocks is ICD programming. The indirect effects of changes in programming practice after the implementation of the 2015 guidelines on optimized ICD programming [16, 17] were therefore examined through analysis of event rates per 100 person-years for ICD shocks in those implanted before and after 2015, adjusted for the above-described risk factors and tested for statistically significant differences through Cox proportional hazard regression analysis. Moreover, the performance of shock prevention algorithms and default programming may vary considerably between ICD manufacturers [19, 20] which is why we examined event rates per 100 person-years between manufacturers in the same fashion as above described. To compare the effects on outcomes by device manufacturer, sensitivity analyses were carried out using the Cox proportional hazard regression model.

A two-sided *p*-value of 0.05 or below was considered statistically significant for the analyses. All data handling and analyses were performed using R version 4.2.2 (2022–10-31).

### Data approval

The study was carried out in accordance with the Declaration of Helsinki. Data approval was obtained by the Danish Data Protection Agency (R-20073406). In Denmark, registry-based studies using pseudo-anonymized data, do not require ethical approval.

## Results

### Study population

A total of 2998 patients were included and of them, 2950 (98.4%) had available information about PP and SP status. The mean age was 61.8 ± 12.7 years, 20.0% were female, 73.0% were ICD carriers versus 27.0% with CRT-D, and 47.1% had a device implanted for SP. Baseline characteristics are summarized in Table 1.

**Table 1:** Baseline characteristics

	Baseline characteristics			
Characteristic^a^	Overall,* N* = 2,950^b^	PP, *N* = 1,539^b^	SP, *N* = 1,411^b^	*p*-value^c^
Age (years)	62 ± 13	63 ± 12	60 ± 13	< 0.001
Male sex	2352 (80%)	1226 (80%)	1126 (80%)	> 0.9
LVEF (%)	30 (25, 45)	25 (20, 30)	45 (30, 55)	< 0.001
NYHA				< 0.001
*I*	726 (25%)	142 (9.3%)	584 (42,5%)	
*II*	1640 (56.6%)	961 (63%)	679 (49,5%)	
*III*	522 (18%)	416 (27.2%)	106 (7.7%)	
*IV*	11 (0.4%)	7 (0.5%)	4 (0.3%)	
Device type				< 0.001
*ICD*	2156 (73%)	906 (59%)	1250 (89%)	
*CRT-D*	794 (27%)	633 (41%)	161 (11%)	
Indication				< 0.001
*ARVC*	28 (1.0%)	5 (0.3%)	23 (1.7%)	
*Channelopathy*	86 (3.0%)	22 (1.5%)	64 (4.7%)	
*Congenital heart disease*	32 (1.1%)	2 (0.1%)	30 (2.2%)	
*Dilated cardiomyopathy*	521 (18%)	343 (23%)	178 (13%)	
*Hypertrophic cardiomyopathy*	104 (3.6%)	65 (4.3%)	39 (2.8%)	
*Idiopathic VF*	118 (4.1%)	0 (0%)	118 (8.6%)	
*IHD*	1880 (65%)	1031 (68%)	849 (62%)	
*Other cardiomyopathy*	116 (4.0%)	47 (3.1%)	69 (5.0%)	
Previous PCI	1043 (36%)	605 (40%)	438 (32%)	< 0.001
Previous CABG	717 (24%)	365 (24%)	352 (25%)	0.3
BMI category				0.094
*Underweight*	45 (1.6%)	20 (1.4%)	25 (2%)	
*Normal*	965 (34.4%)	481 (32.6%)	484 (36%)	
*Overweight*	1102 (39.2%)	580 (40%)	522 (39%)	
*Obese*	697 (24.8%)	387 (26%)	310 (23%)	

^a^*ARVC* arrhythmogenic right ventricular cardiomyopathy, *BMI* body mass index, BMI category: underweight (BMI < 18.5), overweight (BMI 25–29.9), obese (BMI ≥ 30.0), normal weight (BMI 18.5–24.9); *CABG* coronary artery bypass graft, *CRT-D* Implantable Cardioverter Defibrillator with cardiac resynchronization therapy, *HF* heart failure, *ICD* Implantable Cardioverter Defibrillator, *IHD* ischemic heart disease, *LVEF* left ventricular ejection fraction, *NYHA* New York Heart Association Functional Class, *PP* primary prevention, *PCI* percutaneous coronary intervention, *SP* secondary prevention, *VF* ventricular fibrillation

^b^Mean ± SD; *N* (%);Median (Interquartiles)

^c^Wilcoxon rank sum test; Pearson’s chi-squared test; Student *t*-test

During a median follow-up of 4.3 (IQR 2.1–7.4) years, 711/2998 (23.7%) died before any ICD shock, and 426/2998 (14.2%) experienced incident either appropriate or inappropriate shock, 364 (12.1%) experienced appropriate and 82 (2.3%) experienced inappropriate shock uncensored of one another. Among recipients of a first shock, 112 (26.3%) died before a second shock, and 138 (37.9%) experienced recurrent either appropriate or inappropriate shock, 133 (36.5%) experienced appropriate recurrent shocks and 8 (2.2%) experienced inappropriate recurrent shocks uncensored of one another. A total of 560 (18.7%) patients experienced appropriate ATP, of which 41 (7.3%) occurred after the first ICD shock and 243 (43.4%) occurred in patients who never had a shock. Most ATPs occurred in two of the manufacturers. See Table 2 for details on the distribution of ATP.

**Table 2:** Distribution of ATP^a^

		No ICD shock	Any incident ICD shock	Incident appropriate ICD shock	Incident inappropriate ICD shock	Total^b^
Appropriate ATP (*N* = yes (%))		243 (43.4%)	317 (56.6%)	304 (54.3%)	30 (5.4%)	560
	Male sex	194 (79.8%)	262 (82.6%)	250 (82.2%)	26 (86.7%)	456 (81%)
	Female sex	49 (20.2%)	55 (17.4%)	54 (17.8%)	4 (13.3%)	104 (18.6%)
	PP	87 (36.3%)	109 (35.3%)	107 (47.3%)	7 (25%)	196 (35%)
	SP	153 (63.7%)	200 (64.7%)	119 (52.7%)	21 (75%)	353 (63%)
	CRT-D	45 (18.5%)	61 (19.2%)	57 (18.8%)	7 (23.3%)	106 (18.9%)
	Previous revascularization	121 (50.0%)	159 (50.6%)	153 (50.8%)	13 (44.8%)	280 (50%)
Manufacturer						
	I	125	136	131	13	136
	II	70	123	115	15	123
	III	24	30	30	-	30
	IV	24	27	27	0	27
ATPs that occurred after incident shock			41/317 (12.9%)	33/304 (10.9%)	9/30 (30%)	41 (7.3%)

^a^Distribution of the total of 560 patients that received ATP during follow-up, split between patients receiving no ICD shock versus those that received incident ICD shock

^b^The columns with shock may overlap, explaining why the sum is not equal to the number of patients with shock

Abbreviations: *ATP* anti-tachycardia pacing, *CRT-D* cardiac resynchronization therapy-defibrillator, *ICD* Implantable Cardioverter defibrillator; previous revascularization, history of either percutaneous cardiac intervention (PCI) or coronary artery bypass graft (CABG)

A total of 62/2998 (2.1%) patients only experienced inappropriate ICD shock, without ever experiencing an appropriate shock, of which five (1.2%) further experienced only an inappropriate recurrent shock (one in PP and four in SP). Out of these 62 patients, five (9.7%) patients experienced device-related complication and 13 (21.0%) experienced appropriate ATP, none of which occurred in patients with two inappropriate shocks. There were no patients with only inappropriate shock that experienced both device-related complications and appropriate ATP. The most common reason for inappropriate shock, regardless of inappropriate shocks with or without either appropriate shocks and/or appropriate ATP, was atrial fibrillation (AFib) or atrial flutter (AFL) for the population overall in PP and in SP, as can be seen in Supplementary Table S1.

Regarding device-related complications, the most frequently seen generator complication was systemic infection/endocarditis (13%). The most common lead-derived complication was lead displacement (37%). All device-related complications can be seen in Supplementary Table S2.

With regards to NNT and NNH analyses, with the assumption of an absolute risk at 0% in the comparator group, the absolute risk difference was 10.3% (10.3–0%), and thus a NNT of 10 (100/10.3 = 10). Consequently, ten patients needed to be treated with ICD to prevent that one patient has VT/VF that goes untreated, while 47 patients needed to be treated with ICD to cause one inappropriate shock during approximately 4 years of follow-up. When adding serious device-related complications together with inappropriate shock, the NNH fell to 14. Between prevention groups, the NNT was 17 in PP and 7 in SP while the NNH was 66 and 35 in PP and SP, respectively. Overall NNH was higher than NNT within groups. All NNT and NNH results can be seen in Supplementary Table S3.

Analysis on heart failure medication and antiarrhythmic drug use in the 90 days prior to shock as well as corrective measures after incident appropriate and inappropriate shock was performed in a subpopulation experiencing the first shock occurring after January 1, 2017 (*n* = 209), of which 183 and 26 had incident appropriate and inappropriate shock, respectively. Medication use with regard to treatment of heart failure or antiarrhythmic drugs did not differ between those with appropriate and inappropriate shock, whereas the use of diuretics, ACE inhibitors, and aldosterone antagonists was significantly higher in PP compared to SP (Supplementary Table S4). Among the 183 with incident appropriate shock, 18 (9.8%) had ablation for VT performed, and 2 (1.1%) for ventricular extrasystoles, and for 50 (27.3%) patients reprogramming was carried out. The most common change in medication was betablockers and class III antiarrhythmic drug (AAD) regimes. In the 26 with inappropriate incident shock occurring after January 1, 2017, one (3.8%) patient received ablation for AFib/AFL in proximity after the shock, 15 (57.7%) had reprogramming performed and the most common change in AAD was betablocker regime in the month after shock. All changes in AAD regimen after shocks for this subpopulation are listed in Supplementary Table S5a + b.

### Rates of incident and recurrent shock in patients with primary or secondary prevention ICD

The 5-year cumulative incidence of appropriate shock was 11.0% [95% CI 9.9; 12.2] for the entire study cohort and 6.7% [95% CI 5.4; 8.0] in PP versus 15.5% [95% CI 13.5; 17.4] in SP (*p* < 0.001), with annualized event rates estimated to 1.49 [95% CI 1.24; 1.78] and 3.28 [95% CI 2.87; 3.74] per 100 person-years, respectively. Event rates were significantly different between PP and SP for any shock (*p* < 0.001), appropriate shock (*p* < 0.001), and inappropriate shock (*p* = 0.0018), as presented in Table 3. Of the 426 cases with incident shock, 109 (25.6%) happened within 6 months of implantation, 168 (39.4%) within the first year, and 354 (83.1%) within 5 years of implantation.

**Table 3:** Event rates of appropriate and inappropriate ICD shocks per 100 person-years (95% CI)

	Appropriate or inappropriate shock therapy			Appropriate shock therapy			Inappropriate shock therapy		
	Overall	PP	SP	Overall	PP	SP	Overall	PP	SP
Incident ICD shock	2.97 (2.70–3.27)	1.89 (1.59–2.22)	4.23 (3.74–4.77)	2.34 (2.11–2.59)	1.49 (1.24–1.78)	3.28 (2.87–3.74)	0.49 (0.39–0.61)	0.33 (0.22–0.48)	0.65 (0.48–0.85)
Recurrent ICD shock	11.24 (9.44–13.28)	10.05 (7.11–13.80)	11.59 (9.39–14.15)	10.57 (8.85–12.53)	9.54 (6.72–13.15)	10.84 (8.74–13.29)	0.46 (0.20–0.92)	0.61 (0.12–1.77)	0.42 (0.14–0.98)

*CI* confidence interval, *ICD* implantable cardioverter defibrillator, *PP* primary prevention, *SP* secondary prevention

In patients with the first appropriate or inappropriate shock, the 5-year cumulative incidence of receiving a second appropriate shock was 33.5% [95% CI 28.7; 38.4] in the population as a whole and 27.7% [95% CI 19.8; 35.6] in PP versus 36.4% [95% CI 30.2; 42.7] in SP (*p* = 0.063). Event rates per 100 person-years were 10.57 [95% CI 8.85; 12.53] and 0.46 [95% CI 0.20; 0.92] for recurrent appropriate and inappropriate shocks, respectively. The recurrent event rate was only significantly different between PP and SP for any recurrent shock (*p* < 0.044), but not for recurrent appropriate nor inappropriate shock (*p* = 0.063 and *p* = 0.91, respectively).

When splitting event rates per 100 person-years by the four manufacturers used in our study population, there was only a significant difference between manufacturer II and I for incident appropriate shock. The rates are presented in Supplementary Fig. S1a and S1b. During follow-up, a total of 628 (20.9%) patients had a change of generator, of which 557 were battery changes and 71 were device up- or downgrades. In these, a different generator manufacturer was chosen during the procedures in 89 and 29 of patients, respectively.

Furthermore, 44 (1.5%) went through a second generator procedure, of which 35 were battery changes and 9 were up- or downgrades, respectively. The same manufacturer was then most often continued during battery change. For the few patients who had three generator procedures after implantation, none received a new generator from a different manufacturer.

Of the 138 cases with recurrent shock, 70 (50.7%) happened within 6 months of the first shock, 81 (58.7%) within the first year, and 132 (95.7%) within 5 years of the first shock. The median follow-up time from the first shock was 1.93 (IQR 0.44–4.7) years.

**Fig. 1:**
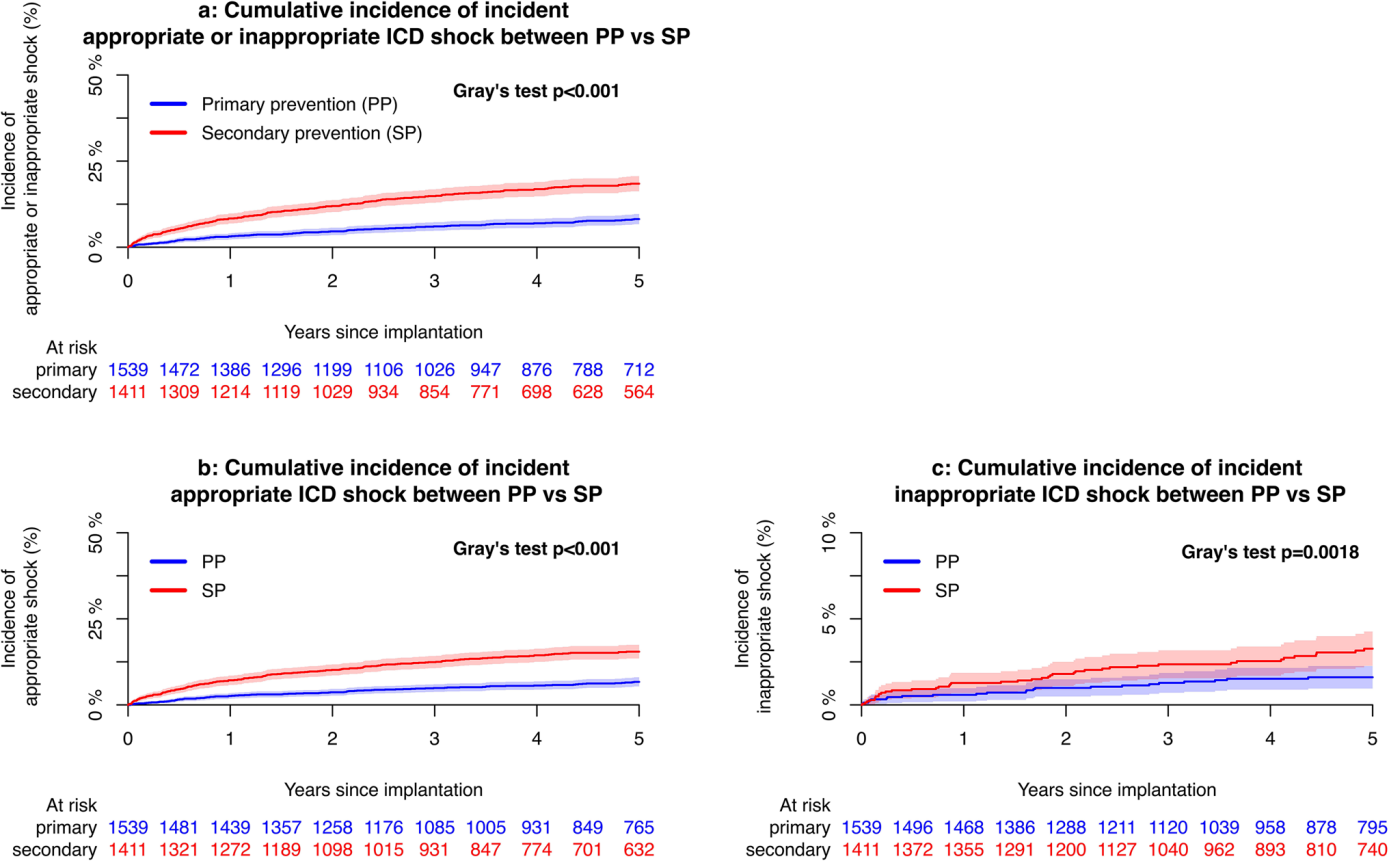
Cumulative incidence of incident ICD shock therapies between PP versus SP Abbreviations: ICD, implantable cardioverter defibrillator; PP, primary prevention; SP, secondary prevention

**Fig. 2:**
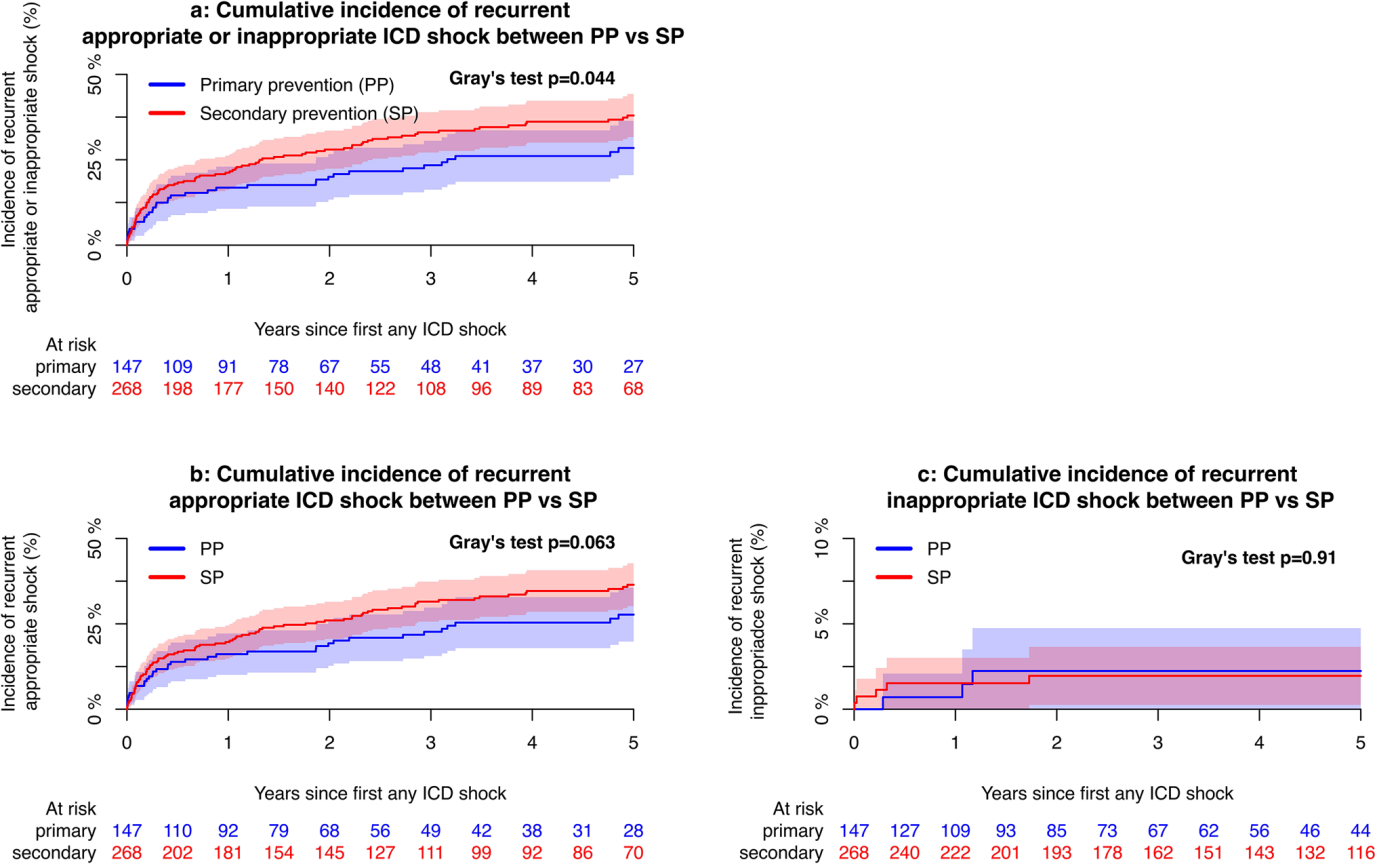
Cumulative incidence of recurrent ICD shock therapies between PP versus SP Abbreviations: ICD, implantable cardioverter defibrillator; PP, primary prevention; SP, secondary prevention

Cumulative incidence curves of appropriate and inappropriate incident and recurrent shock are displayed in Figs. 1a to 2c. Event rates per 100 person-years of incident appropriate, inappropriate, and any shock declined over time when comparing those implanted before and after 2015 as seen in Supplementary Fig. S2a. Changes in rates for recurrent shock before and after 2015 did not reach statistical difference between groups as displayed in Supplementary Fig. S2b. With regard to NNT and NNH for patients implanted between 2010 and 2015, eight patients needed to be treated with ICD to prevent one patient from having VT/VF that goes untreated, while 36 patients needed to be treated with ICD to cause one inappropriate shock during approximately 4 years of follow up. For patients implanted after 2015, these numbers increased to 15 and 86, respectively.

### Risk factors for incident ICD shock

Risk factors associated with reduced risk of *incident shock* in the overall population that all met the current guideline-based criteria for ICD implantation were female sex, CRT-D, and revascularization for ischemic heart disease (HR 0.69 [95% CI 0.52; 0.91] *p* = 0.01, HR 0.71 [95% CI 0.54; 0.94] *p* = 0.0016, and HR 0.78 [95% CI 0.63; 0.96] *p* = 0.019, respectively), no significant interactions were observed between these risk factors and the prevention groups (*p* for interaction = 0.50, 0.17, and 0.81, respectively).

Female sex showed an association with reduced risk of *incident appropriate shock* (HR 0.68 [95% CI 0.49; 0.92] *p* = 0.014), without any significant interaction between prevention groups and female sex (*p* for interaction = 0.97). Contrary, having a CRT-D showed a significant interaction with prevention groups (*p* for interaction 0.044), where having a CRT-D versus ICD was associated with a reduced risk of appropriate shock only in SP (HR 0.43 [95% CI 0.24; 0.77] *p* = 0.0048).

**Fig. 3:**
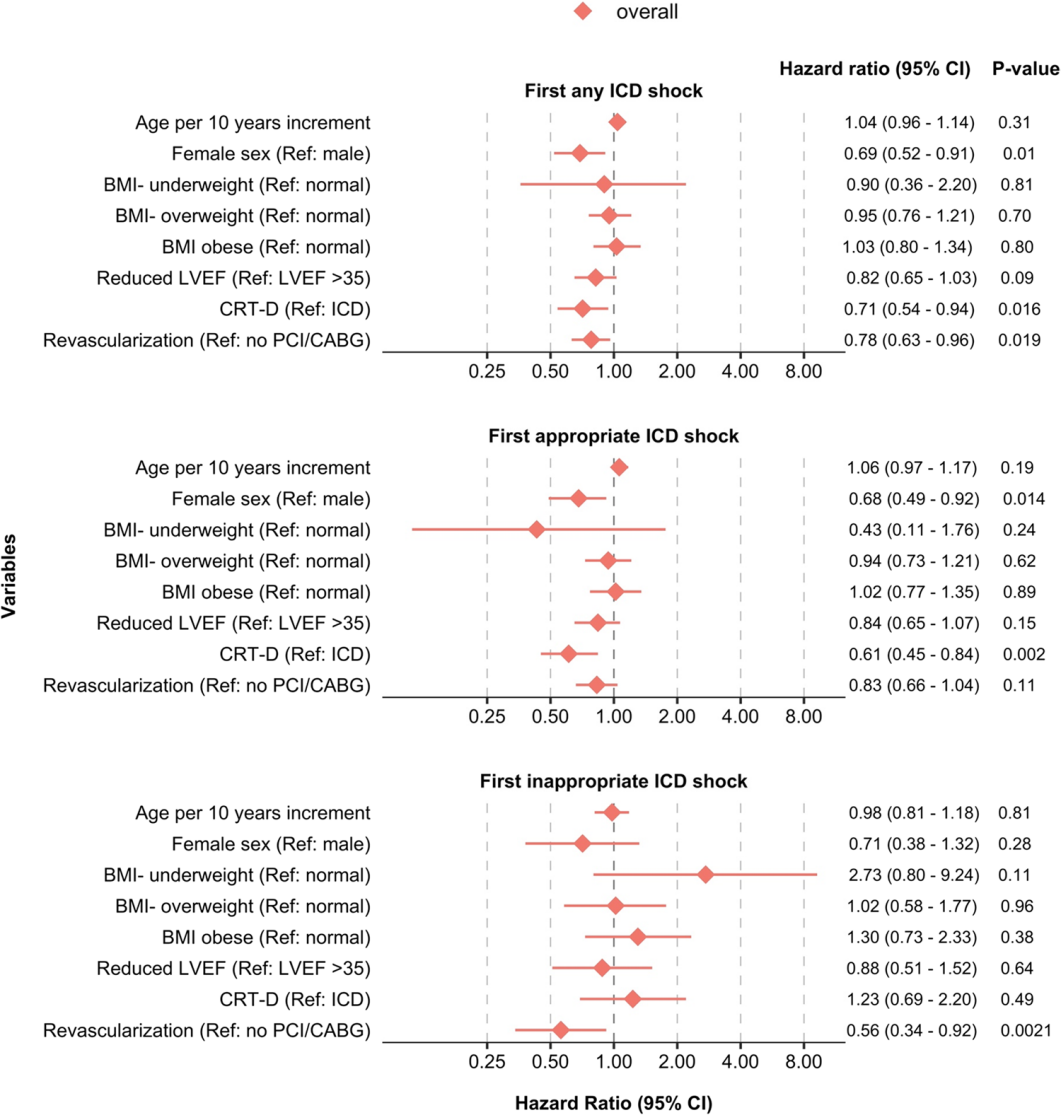
Multivariable models on risk factors for ICD therapy This forest plot shows multivariable Cox proportional hazard regression models for time to first of each outcome, for primary and secondary prevention together. Results are given as HR [95% CI], *P*, and each model was adjusted for covariates as indicated by the given estimates. Abbreviations: BMI, body mass index; BMI category: underweight (BMI < 18.5 kg/m^2^), overweight (BMI 25–29.9), obese (BMI ≥ 30.0), normal weight (BMI 18.5–24.9); CABG, coronary artery bypass graft; CRT-D, Implantable Cardioverter Defibrillator with cardiac resynchronization therapy; ICD, Implantable Cardioverter Defibrillator; LVEF, left ventricular ejection fraction (Reduced LVEF was defined as LVEF ≤ 35%); PCI, percutaneous coronary intervention

Previous revascularization showed an association with reduced risk of first-time *inappropriate shock* overall (HR 0.56 [95% CI 0.34; 0.92] *p* = 0.021). With regard to BMI, there was a significant interaction between prevention groups (*p* for interaction = 0.0047) where BMI > 30 compared to normal weight was associated with an increased risk of inappropriate shock only in SP (HR 2.85 [95% CI 1.30; 6.24] *p* = 0.0088). Age was not associated with any outcomes. For all results on incident shock, see Fig. 3.

Sensitivity analyses for incident shocks, adjusted for manufacturer, rendered similar results as the above and are displayed in Supplementary Table S6.

### Risk factors for recurrent ICD shock following first appropriate or inappropriate shock

In patients with previous shock, a risk factor associated with reduced risk of *recurrent shock* was CRT-D in the overall cohort (HR 0.54 [95% CI 0.30; 0.96] *p* = 0.036), without any significant interaction between prevention groups (*p* for interaction 0.93). There was a significant interaction between prevention groups and revascularization status (*p* for interaction = 0.016), where prior revascularization was associated with increased risk of recurrent shock (HR 2.93 [95% CI 1.42; 6.05] *p* = 0.0036) in PP only, compared to those without prior revascularization.

For the endpoint of *recurrent appropriate shock,* having a CRT-D was associated with a reduced risk of an event (HR 0.53 [95% CI 0.29; 0.96] *p* = 0.036). There was a significant interaction between prevention groups and revascularization status (*p* for interaction = 0.014), where prior revascularization versus no revascularization was associated with an increased risk of recurrent appropriate shock (HR 3.32 [95% CI 1.57; 7.02] *p* = 0.0017) in PP only.

**Fig. 4:**
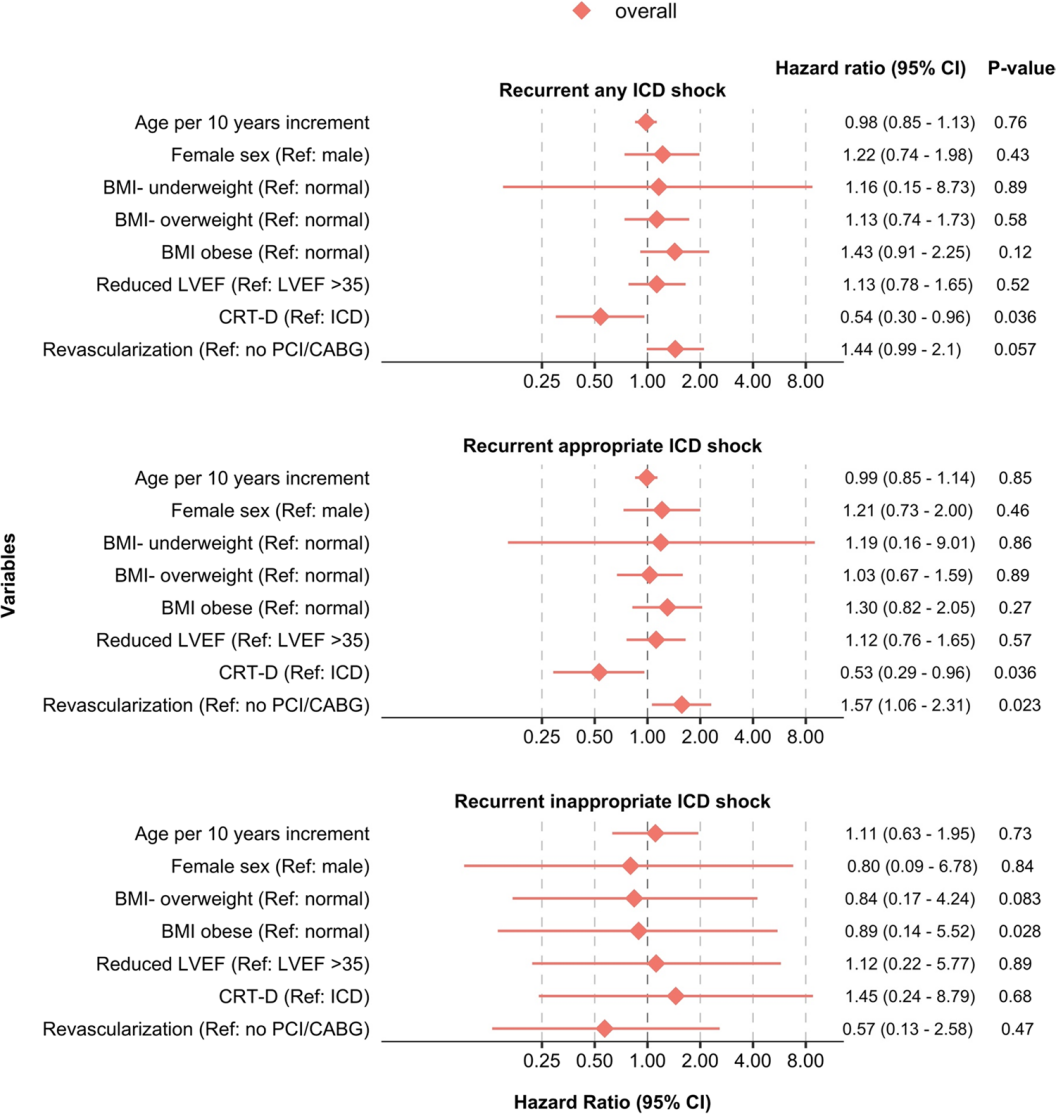
Multivariable models on risk factors for ICD therapy following first ICD shock This forest plot shows multivariable Cox proportional hazard regression models for the time from the first to second of each outcome, for primary and secondary prevention together. Results are given as HR [95% CI], *P*, and each model was adjusted for covariates as indicated by the given estimates. Abbreviations: BMI, body mass index; BMI category: underweight (BMI < 18.5 kg/m^2^), overweight (BMI 25–29.9), obese (BMI ≥ 30.0), normal weight (BMI 18.5–24.9); CABG, coronary artery bypass graft; CRT-D, Implantable Cardioverter Defibrillator with cardiac resynchronization therapy; ICD, Implantable Cardioverter Defibrillator; LVEF, left ventricular ejection fraction (reduced LVEF was defined as LVEF ≤ 35%); PCI, percutaneous coronary intervention

Age, female sex, and reduced LVEF did not show significant associations with recurrent shocks. All results for recurrent therapies can be seen in Fig. 4.

Sensitivity analyses for recurrent shocks, adjusting for manufacturer, rendered similar results as the above, except for device type which did not reach statistical significance neither for recurrent any shock or recurrent appropriate shock in the sensitivity analyses. The results of the sensitivity analyses are presented in Supplementary Table S7.

## Discussion

We conducted a large single-center cohort study of contemporary ICD recipients with a median follow-up of more than 4 years and made the following key findings: (1) One in eight patients with ICD implanted according to contemporary guidelines received a first-time appropriate shock, and even lower in PP. (2) The rates of inappropriate shock were generally much lower, around fivefold lower, than for appropriate shock, although not negligible, and have decreased over time. (3) The rate of shock increased significantly following incident shock, especially in PP. (4) Few clinical variables were associated with events and thus possible benefit from ICD therapy.

The novelty of our study lies within the contemporary cohort and the examination of recurrent ICD therapy and the associated risk factors, further discussed below.

### ICD therapy rates over time

The rates of mortality and ICD shock today are considerably lower than in the initially carried out Antiarrhythmics Versus Implantable Defibrillators (AVID) Trial, Multicenter Automatic Defibrillator Implantation Trial (MADIT), and Sudden Cardiac Death in Heart Failure Trial (SCD-HeFT) studies [21–23], as well as other ICD studies of the early 2000s [24–26]. During that time, a PP ICD cohort demonstrated that 7.8% and 2.6% experienced appropriate and inappropriate shock, respectively, during a mean follow-up time of approximately 2 years, and another study from the same time period demonstrated a 1-year cumulative incidence of any shock of 33.3% in an SP population [24, 26], all numbers distinctively higher than those found in our study. This downward trend is likely due to temporal changes in the ICD population. Advances in medical treatment of especially heart failure and revascularization strategy may have contributed to a decrease in outcomes and thereby a decline in benefit from implantation. Further, improvements in programming and increasing use of ATP may have altered the risk of shock or inappropriate therapy over time [3–5, 27]. Concerns have been raised that optimizing ICD programming to reduce ICD therapy might lead to failed treatment of ventricular arrhythmias. A study addressing this evaluated the outcomes of PP ICD patients in centers with varying adherence to ICD programming guidelines. Patients in centers with high guideline adherence experienced significantly lower rates of ICD therapy, primarily due to reduced ATP, without any difference in mortality during follow-up. These findings suggest that adherence to ICD programming guidelines significantly benefits ICD therapy rates, underscoring the importance of strict guideline implementation to optimize patient outcomes [28].

Despite not having direct data on the programming of devices in this study, our results imply a downward trend in event rates after the implementation of the 2015 guidelines on optimized ICD programming [16, 17] regardless of manufacturers, in which standard settings might differ. Furthermore, the harm is decreasing (higher NNH), which could be due to better concomitant medicine and better ICD algorithms and programming, while the benefit is also decreasing (higher NNT), which could be due to a lower absolute risk of ventricular arrhythmia. Yet, overall NNH remained higher than NNT in subgroups. In pursuit of selecting the most suitable candidates for ICD implantation, and optimizing their follow-up treatment, these differences in the contemporary ICD population compared to that of the initial studies are important. The temporal changes are likely applicable to have consequences for inappropriate and recurrent ICD shock rates as well [29].

Especially low are the rates of incident therapy in PP, where LVEF to date still plays a dominant role in current guidelines of ICD candidate selection [30, 31]. Our results did not find reduced LVEF to impact the risk of incident or recurrent ICD shocks, and the dilemma of most SCD occurring in patients with LVEF > 35% remains [32]. There were in general much lower rates of inappropriate shock than for appropriate shock, although not negligible with an event rate of inappropriate shocks of 0.49 [95% CI 0.39; 0.61] per 100 person-years. Especially the ICD recipients who do not receive any subsequent appropriate therapy including no appropriate ATP, are important to identify, as the absence of appropriate therapy combined with the potential presence of other device-related complications, corresponds to patients only experiencing possible harm from their device rather than the anticipated benefit that led to the initial device implantation. Merely one in five patients with only inappropriate shock experienced appropriate ATP and hence may have had use of ICD implantation within the study period. Later, the need for ICD therapy cannot be ruled out. The exact definition of appropriate ICD implantation is complex and not only described by the presence of shock and ATP. Contrary, pharmaceutical treatment of underlying heart conditions, especially heart failure, as well as programming improvements, and increased ATP use alter patient longevity and the number of ICD therapies given throughout a patient’s life.

### Recurrent ICD therapy

Our results imply that once a patient’s ICD has been used for shock therapy, the likelihood of recurrent ICD shock therapy increases noticeably. After a first shock, extra caution should be considered in PP ICD carriers as the event rate per 100 person-years for appropriate recurrent shock increased over sixfold in PP ICDs compared to a threefold increase in SP ICDs. This increased VA incidence and increased mortality in PP rather than in SP patients after a first shock has previously been described in a study by Zhou et al. [33]. Recurrent therapy is a problem [34, 35] and complementary to optimized ICD candidate selection, knowledge of which ICD carriers are more prone to recurrent ICD therapies would further augment the benefit-risk stratification process.

The rates for recurrent inappropriate shock are also not negligible and show the same pattern, with an almost twofold increase in PP ICDs compared to an actual decrease in SP ICDs for recurrent inappropriate shock.

Moreover, in our population, 62 patients only experienced inappropriate ICD shock, of which 5 further experienced an inappropriate recurrent shock. Most of these inappropriate recurrent shocks occurred in SP. In patients with recurrent inappropriate shock without appropriate shock, no one experienced appropriate ATP.

Understanding recurrent ICD therapy patterns is crucial for discussions about the potentially sustained necessity of the device, especially at the time of generator replacement long after the initial implantation. One small-scale study compared 29 (16.8%) patients who, at the time of replacement, did not have an ongoing guideline indication for a PP ICD with those that did [36]. An active indication was defined as having received appropriate ICD therapy or an LVEF ≤ 35% at the time of replacement. Lacking a theoretical indication for ICD at replacement was associated with a lower risk of mortality and incident VA. Another study with a PP ICD population showed that these patients had increased all-cause mortality (31% versus 15%, *p* = 0.005) despite a subsequent improvement in LVEF to > 35% but the number of appropriate therapies received did not significantly differ for patients with continued LVEF below the cutoff of 35% compared to those with improvement in their LVEF [37].

Lastly, knowledge of the incidence and risk factors for recurrent shocks may potentially also be extrapolated to broader use, e.g., in determining the benefit of VT ablation among those experiencing incident shocks, as recurrence of VT after ablation remains a significant clinical problem [38, 39].

### Predictors of ICD shock

The derived use of risk factors for incident and recurrent ICD shock are distinctively different, as the former can be used for ideal ICD candidate selection, whereas the latter can be used for optimization of treatment for those that have received an ICD [1, 31].

Our study found associations of some conventional risk factors that could add value during the ICD candidate selection, in the form of female sex and those with previous revascularization to have a lower risk of incident any shock, while female sex was also associated with lower risk of incident appropriate shock. Having a CRT-D was also a factor in lowering the risk of incident any shock and incident appropriate shock, likely due to improvements in LVEF [40, 41].

The association of female sex with reduced risk of shock was previously demonstrated in a meta-analysis of PP and a recent review examining both prevention groups [42, 43]. Females with IHD and a SP ICD had a significantly lower incidence of VA and SCD, and noticeably fewer appropriate shocks compared to men, in line with our results [43, 44]. Due to common female underrepresentation in larger trials, adequately sized randomized, prospective studies in female populations are therefore warranted. Our results showed an association between IHD with previous revascularization and lower risk of incident shock and inappropriate shock in the overall population, in line with what has been shown previously in a mixed prevention cohort [45], and may be due to an arrhythmogenic substrate potentially profiting the most from revascularization [46]. Contrary, a lack of association with aborted sudden cardiac death was shown in patients with severely reduced LVEF and known ischemic disease with viable myocardium, when comparing the randomized arms of optimal medical treatment with or without concomitant PCI [47].

Regarding optimization of ICD patient treatment, hence considering risk factors for recurrent ICD shock, previous revascularization was associated with an increased risk of recurrent any and recurrent appropriate shock in PP, and BMI > 30 was associated with an increased risk of recurrent inappropriate shock in SP.

Our finding of an association between previously performed revascularization in patients with PP ICD and increased risk of recurrent shock suggests that IHD with a greater extent of atherosclerosis increases the tendency for recurrent arrhythmia and generally worsens the prognosis in patients with a PP ICD [48, 49]. Noteworthy, the VANISH study looking at long-term revascularization effects, regardless of having an ICD, found no associated benefit between previous revascularization and ventricular arrhythmia [50].

In the literature, recurrent ICD therapy is scarcely examined but is mentioned with regard to ICD driving guidelines, as the risk of recurrent ICD therapy affects the time when a patient is again deemed suitable for driving [7]. In a large cohort study by Merchant et al., device programming, i.e., the number of VT and VF therapy zones and lower shock delivery heart rate threshold were both significantly associated with the likelihood of recurrent ICD shock. Implantation year and single or dual chamber device did not show an association. Factors that were associated with the time from incident to recurrent ICD shock were younger implantation age, female sex, a lower programmed shockable detection heart rate, lower LVEF, and SP device [7]. Their study had 14,230 (19.4%) of the population that experienced at least one ICD shock (appropriate and inappropriate combined), a relatively close frequency to that of our study of 14.2%. The incidence of recurrent ICD shock was 37.6% (*n* = 5357) in the period of 2008–2013 [7] comparable to that in our study of 37.9%. No intervention performed after the first inappropriate therapy was shown to be the sole risk factor for subsequent recurrent inappropriate therapy [51].

In conclusion, conventional risk factors did not render sufficient results to convincingly improve neither ICD candidate selection nor follow-up strategies for ICD patients. The improved utility of clinical risk assessment and stratification scores demand further exploration of recurrent ICD therapy beyond these conventional risk factors. The use of imaging, artificial intelligence, and modern (bio)markers has shown promise [52–54].

## Limitations

First, this was an observational study, and therefore no causal inference could be made from our findings. Second, we categorized IHD based on previous revascularization. Consequently, patients with milder degrees of atherosclerosis, not demanding revascularization, have for the purpose of our analyses been labelled as non-IHD patients. Third, the entering of appropriate and inappropriate therapy into the registry was done in a prospective fashion by the treating physician or device technician but may be erroneously not reported, potentially leading to an underrepresentation of the number of actual events. Fourth, due to this study being a single-center study, there might be international differences that decrease the generalizability to other ICD populations. Fifth, serial echocardiographic measurements were lacking, and the analyses could not account for potential changes in left ventricular ejection fraction or other markers leading up to or following ICD shock. Lastly, the decrease in need for ICD shocks over time seen in our study should cautiously be concluded as equal to decreasing ICD benefit as the cause is likely multifactorial, including better overall management of the underlying cardiac conditions, as well as a reflection of improved device programming, with longer detection times, higher rate cutoffs and increased use of ATP to reduce shocks. The benefit from an ICD is a combination of appropriate therapy, lack of inappropriate therapy, and absence of any other device-related side effects.

## Conclusion

This large, contemporary, single-center study of patients who met current guideline-based criteria for ICD implantation found a lower incidence of first-time shock compared to the earlier, guideline-generating studies. One in eight received an appropriate ICD shock during a median follow-up of 4.3 years and one-third of those experienced recurrent appropriate shock. Recurrent event rates increased most in PP compared to SP for recurrent appropriate and inappropriate shock. Previous revascularization showed an association with an increased risk of recurrent any shock and recurrent appropriate shock in PP. All in all, few clinical variables showed potential in identifying patients with benefit from ICD.

## Supplementary Information

Below is the link to the electronic supplementary material.

Supplementary file1 (DOCX 553 KB)

## Data Availability

Data are not publicly available due to the sensitive nature of the research. Relevant data can be made available upon reasonable request.
