# Supplementary material for: Incidence and risk factors for first and recurrent ICD shock therapy in patients with an implantable cardioverter defibrillator
Source: J Interv Card Electrophysiol. 2024 Aug 21;68(1):125–39. doi: 10.1007/s10840-024-01873-0 (PMC11832632; doi:10.1007/s10840-024-01873-0)
Supplement: Supplementary file 1 — Supplementary file1 (DOCX 553 KB) [file 10840_2024_1873_MOESM1_ESM.docx]

**Supplementary**

**Article title: Incidence and risk factors for first and recurrent ICD shock therapy in patients with an implantable cardioverter defibrillator**

**Journal**: Journal of Interventional Cardiac Electrophysiology

Authors: Diana My Frodi^1^, Søren Z. Diederichsen^1^, Lucas Yixi Xing^1^, Daniel Camillo Spona^1^, Peter Karl Jacobsen^1^, Niels Risum^1^, Jesper H. Svendsen^1,2^

**Affiliations**:

^1^Department of Cardiology, Copenhagen University Hospital – Rigshospitalet, Copenhagen, Denmark

^2^ Department of Clinical Medicine, Faculty of Health and Medical Sciences, University of Copenhagen, Copenhagen, Denmark

No. supplementary tables: 7

No. supplementary figures: 2

**Address for Correspondence:**

Jesper Hastrup Svendsen

Professor of Cardiology, consultant, MD, DMSc, FESC, FEHRA

Department of Cardiology, Copenhagen University Hospital – Rigshospitalet,

e-mail: jesper.hastrup.svendsen@regionh.dk

**Supplementary Table S1**

**Reasons for inappropriate ICD shock overall and according to prior appropriate ICD shock status**

|  | Overall cohort | | | PP^1^ | | | SP^1^ | | |
| --- | --- | --- | --- | --- | --- | --- | --- | --- | --- |
|  | Incident inappropriate shock regardless of appropriate therapy^2^  (n=82) | Incident inappropriate shocks without prior appropriate shock^2^  (n=69) | Incident inappropriate shock without prior appropriate shock or appropriate ATP^2^  (n=58) | Incident inappropriate shock regardless of appropriate therapy^2^  (n=29) | Incident inappropriate shocks without prior appropriate shock^2^  (n=25) | Incident inappropriate shock without prior appropriate shock or appropriate ATP^2^  (n=22) | Incident inappropriate shock regardless of appropriate therapy^2^  (n=51) | Incident inappropriate shocks without prior appropriate shock^2^  (n=43) | Incident inappropriate shock without prior appropriate shock or appropriate ATP^2^  (n=36) |
| AFib/AFL | 56 (68.3%) | 47 (68.1%) | 39 (67.2%) | 19 (65.6%) | 16 (64.0%) | 14 (63.6%) | 35 (68.6%) | 30 (69.8%) | 25 (69.4%) |
| SVT | 8 (9.8%) | 6 (8.7%) | 4 (6.9%) | 2 (6.9%) | 2 (8.0%) | 1 (4.5%) | 6 (11.8%) | 4 (9.3%) | 3 (8.3%) |
| Lead related problems | 4 (4.9%) | 4 (5.8%) | 4 (6.9%) | 2 (6.9%) | 2 (8.0%) | 2 (9.1%) | 2 (3.9%) | 2 (4.7%) | 2 (5.6%) |
| ICD related problems | 1 (1.2%) | 1 (1.4%) | 1 (1.7%) | 0 (0%) | 0 (0%) | 0 (0%) | 1 (2.0%) | 1 (2.3%) | 1 (2.8%) |
| T-wave oversense | 4 (4.9%) | 3 (4.3%) | 3 (5.2%) | 3 (10.3%) | 2 (8.0%) | 2 (9.1%) | 1 (2.0%) | 1 (2.3%) | 1 (2.8%) |
| Other | 9 (10.9%) | 8 (11.7%) | 7 (12.1%) | 3 (10.3%) | 3 (12.0%) | 3 (13.7%) | 6 (11.7%) | 5 (11.6%) | 4 (11.1%) |
| ^1^ Due to some missingness for prevention type, PP and SP numbers do not equal the overall numbers.  ^2^ n (%)  Abbreviations: AFib/AFL, atrial fibrillation/atrial flutter; ATP, anti-tachycardia pacing; ICD, Implantable Cardioverter Defibrillator, SVT, supraventricular tachycardia | | | | | | | | | |

**Supplementary Table S2: Device related complications during follow up**

| **Generator related complications** | | | | |  | |  |
| --- | --- | --- | --- | --- | --- | --- | --- |
|  | **Overall**, N = 103^b^ | **1**, N = 60^b^ | **2**, N = 43^b^ | | **p-value**^c^ | |  |
| Major: |  |  |  | |  | |  |
| systemic infection/endocarditis | 32 (31%) | 19 (32%) | 13 (30%) | | 0.9 | |  |
| local pocket infection/skin erosion | 13 (13%) | 11 (18%) | 2 (4.7%) | | 0.039 | |  |
| failure - sensing/pacing | 12 (12%) | 5 (8.3%) | 7 (16%) | | 0.2 | |  |
| Ancillary: | | | | |  | |  |
| premature EOL^a^ | 18 (17%) | 10 (17%) | 8 (19%) | | 0.8 | |  |
| failure - generator-other | 3 (2.9%) | 1 (1.7%) | 2 (4.7%) | | 0.6 | |  |
| recall without malfunction | 2 (1.9%) | 1 (1.7%) | 1 (2.3%) | | >0.9 | |  |
| other | 23 (22%) | 13 (22%) | 10 (23%) | | 0.8 | |  |
| **Lead related complications** | | | | | |  | |
|  | **Overall**, N = 353^b^ | **1**, N = 223^b^ | | **2**, N = 130^b^ | | **p-value**^c^ | |
| Major: |  |  | |  | |  | |
| displacement | 132 (37%) | 84 (38%) | | 48 (37%) | | 0.9 | |
| systemic infection/endocarditis | 51 (14%) | 34 (15%) | | 17 (13%) | | 0.6 | |
| local pocket infection/skin erosion | 24 (6.8%) | 20 (9.0%) | | 4 (3.1%) | | 0.034 | |
| Ancillary: | | | |  | |  | |
| high pacing threshold | 40 (11%) | 18 (8.1%) | | 22 (17%) | | 0.011 | |
| oversensing | 19 (5.4%) | 9 (4.0%) | | 10 (7.7%) | | 0.14 | |
| undersensing | 12 (3.4%) | 6 (2.7%) | | 6 (4.6%) | | 0.4 | |
| high impedance | 9 (2.5%) | 7 (3.1%) | | 2 (1.5%) | | 0.5 | |
| insulation failure | 10 (2.8%) | 7 (3.1%) | | 3 (2.3%) | | 0.8 | |
| cardiac perforation | 4 (1.1%) | 1 (0.4%) | | 3 (2.3%) | | 0.14 | |
| conductor break | 4 (1.1%) | 3 (1.3%) | | 1 (0.8%) | | >0.9 | |
| high defibrillation threshold | 3 (0.8%) | 1 (0.4%) | | 2 (1.5%) | | 0.6 | |
| extracardiac stimulation | 2 (0.6%) | 2 (0.9%) | | 0 (0%) | | 0.5 | |
| generator lead interface problem | 1 (0.3%) | 1 (0.4%) | | 0 (0%) | | >0.9 | |
| myopotential inhibition | 1 (0.3%) | 1 (0.4%) | | 0 (0%) | | >0.9 | |
| other | 41 (12%) | 29 (13%) | | 12 (9.2%) | | 0.3 | |
| ^a^Abbreviations= EOL, end of life | | | | | | | |
| ^b^n (%) | | | | | | | |
| ^c^Pearson's Chi-squared test | | | | | | | |

**Supplementary Table S3**

**Numbers needed to treat, and Numbers needed to harm analyses**

|  | **Population as a whole** | **PP** | **SP** | **Implantation before 2015** | **Implantation after 2015** |
| --- | --- | --- | --- | --- | --- |
| **NNT** |  |  | |  | |
|  | 10 | 17 | 7 | 8 | 15 |
| **NNH**^a^ |  |  |  |  |  |
| NNH inapp. shock | 47 | 66 | 35 | 36 | 86 |
| NNH inapp. shock + serious | 14 | 13 | 15 | 13 | 15 |
| NNH inapp. shock + all | 10 | 10 | 11 | 10 | 10 |
| Abbreviations: NNH, number needed to harm; NNT, number needed to treat; PP, primary prevention; SP, secondary prevention.  ^a^NNH calculated by dividing the safety outcome into the three categories inapp. shock (=inappropriate shock), inapp. shock + serious (=inappropriate shock and/or serious device related complications (defined in Supplementary Table S2)) and iapp. shock + all (=inappropriate shock and/or any device related complication (defined in Supplementary Table S2), respectively. | | | | | |

**Supplementary Table S4**

| **Medications in the 90 days prior to first ICD shock from 2017 and onwards** | | | | | | | |
| --- | --- | --- | --- | --- | --- | --- | --- |
|  | **Overall**  N = 209^2^ | **Appropriate shock**  N = 183^2^ | **Inappropriate shock**  N = 26^2^ | **p-value**^3^ | **PP**  N = 69^2^ | **SP**  N = 136^2^ | **p-value**^3^ |
| **AAD class I** | 1 (0.5%) | 1 (0.5%) | 0 (0%) | >0.9 | 0 (0%) | 1 (0.7%) | >0.9 |
| **BB** | 173 (83%) | 152 (83%) | 21 (81%) | 0.8 | 60 (87%) | 109 (80%) | 0.2 |
| **AAD class III** | 30 (14%) | 29 (16%) | 1 (3.8%) | 0.14 | 7 (10%) | 21 (15%) | 0.3 |
| **Calcium antagonist** | 24 (11%) | 21 (11%) | 3 (12%) | >0.9 | 4 (5.8%) | 20 (15%) | 0.061 |
| *Dihydropyridin* | 24 (89%) | 21 (88%) | 3 (100%) |  | 5 (83%) | 19 (90%) |  |
| *Non-dihydropyridin* | 3 (11%) | 3 (13%) | 0 (0%) |  | 1 (17%) | 2 (9.5%) |  |
| **Digoxin** | 23 (11%) | 21 (11%) | 2 (7.7%) | 0.7 | 13 (19%) | 9 (6.6%) | 0.008 |
| **Sinus node inhibitor** | 5 (2.4%) | 5 (2.7%) | 0 (0%) | >0.9 | 5 (7.2%) | 0 (0%) | 0.004 |
| **ACE inhibitor** | 95 (45%) | 85 (46%) | 10 (38%) | 0.4 | 39 (57%) | 54 (40%) | 0.022 |
| **ARB** | 63 (30%) | 56 (31%) | 7 (27%) | 0.7 | 20 (29%) | 41 (30%) | 0.9 |
| **Aldosterone antagonist** | 79 (38%) | 69 (38%) | 10 (38%) | >0.9 | 33 (48%) | 43 (32%) | 0.023 |
| **SGLT2 inhibitor** | 3 (1.4%) | 3 (1.6%) | 0 (0%) | >0.9 | 2 (2.9%) | 1 (0.7%) | 0.3 |
| **Diuretics** | 110 (53%) | 98 (54%) | 12 (46%) | 0.5 | 47 (68%) | 60 (44%) | 0.001 |
| Abbreviations: AAD, antiarrhythmic drug; ACE, angiotensin-converting enzyme inhibitor; ARB, angiotensin II receptor inhibitor; BB, beta blocker; PP, primary prevention; SGLT2 inhibitor, sodium-glucose cotransporter II inhibitor; SP, secondary prevention; | | | | | | | |
| ^2^n (%) | | | | | | | |
| ^3^Pearson's Chi-squared test | | | | | | | |

**Supplementary Table S5**

**Drug management 30 days post-shock**

**a)**

| AAD changes after incident appropriate shock from 2017 and onwards (n=183 patients with appropriate shock) | | | |
| --- | --- | --- | --- |
|  | No change (%) | Change (%) | Not used (%) |
| BB | 99 (54.1) | 60 (32.8) | 24 (13.1) |
| Digoxin | 16 (8.7) | 8 (4.4) | 159 (86.9) |
| Class I AAD | 0 (0) | 5 (2.7) | 178 (97.3) |
| Class III AAD | 16 (8.8) | 50 (27.3) | 117 (63.9) |
| Calcium antagonist | 16 (8.8) | 7 (3.8) | 160 (87.4) |

Abbreviations: AAD, antiarrhythmic drug; BB, betablocker

Change in drug management covers dosage increase/decrease, new prescription, or termination of drug.

**b)**

| AAD changes in the first month after incident inappropriate shock that occurred from 2017 and onwards (n=26 patients with inappropriate shock) | | | |
| --- | --- | --- | --- |
|  | No change (%) | Change (%) | Not used (%) |
| BB | 17 (65.4) | 9 (34.6) | 0 (0) |
| Digoxin | 2 (7.7) | 3 (11.5) | 21 (80.8) |
| Class I AAD | NA | NA | 26 (100) |
| Class III AAD | 1 (3.9) | 3 (11.5) | 22 (84.6) |
| Calcium antagonist | 3 (11.5) | 0 (0) | 23 (88.5) |

Abbreviations: AAD, antiarrhythmic drug; BB, betablocker

Change in drug management covers dosage increase/decrease, new prescription, or termination of drug.

**Supplementary Table S6**

**Sensitivity analysis- Multivariable Cox analysis for incident ICD shock adjusted for manufacturer**

|  | Incident any shock | P for interaction | Incident appropriate shock | P for interaction | Incident inappropriate shock | P for interaction |
| --- | --- | --- | --- | --- | --- | --- |
| Age per 10-year increment | 1.05 [0.96;1.14] 0.290 | 0.869 | 1.07 [0.97;1.17] 0.160 | 0.803 | 0.97 [0.81;1.17] 0.782 | 0.147 |
| Female sex (Ref: Male) | 0.69 [0.52;0.92] 0.0108* | 0.487 | 0.68 [0.49;0.93] 0.0170* | 0.925 | 0.70 [0.38;1.29] 0.253 | 0.206 |
| BMI- obese (Ref: normal) | 1.03 [0.79;1.34] 0.830 | 0.238 | 1.02 [0.77;1.35] 0.901 | 0.806 | 1.29 [0.72;2.33] 0.397 | 0.0071* |
| BMI- overweight (Ref: normal) | 0.97 [0.77;1.23] 0.833 | 0.238 | 0.95 [0.74;1.23] 0.710 | 0.806 | 1.06 [0.61;1.85] 0.836 | 0.0071* |
| BMI-underweight (Ref: normal) | 0.89 [0.36;2.19] 0.800 | 0.238 | 0.43 [0.11;1.76] 0.241 | 0.806 | 2.64 [0.78;8.97] 0.119 | 0.0071* |
| Reduced LVEF (Ref: LVEF >35) | 0.82 [0.65;1.03] 0.0923 | 0.904 | 0.83 [0.65;1.05] 0.124 | 0.680 | 0.93 [0.53;1.62] 0.795 | 0.677 |
| Device (ref: ICD) | 0.70 [0.53;0.94] 0.0168* | 0.153 | 0.64 [0.46;0.88] 0.00579* | 0.0320* | 0.99 [0.54;1.80] 0.978 | 0.520 |
| Revascularization  (ref: no PCI/CABG) | 0.79 [0.64;0.98] 0.0283* | 0.799 | 0.84 [0.67;1.05] 0.127 | 0.683 | 0.59 [0.36;0.97] 0.0377* | 0.971 |

Multivariable cox proportional hazard regression models for time to first of each outcome, for primary and secondary prevention together. P for interaction with primary and secondary prevention.

Results are given as HR [95% CI], P, and each model is adjusted for covariates as indicated by the given estimates, as well as manufacturer.

Abbreviations: BMI, body mass index; BMI category: underweight (BMI <18.5), overweight (BMI 25-29.9), obese (BMI ≥ 30.0), normal weight (BMI 18.5-24.9); CABG, coronary artery bypass graft; CRT-D, Implantable Cardioverter Defibrillator with cardiac resynchronization therapy; ICD, Implantable Cardioverter Defibrillator; LVEF, left ventricular ejection fraction (Reduced LVEF was defined as LVEF ≤35%); PCI, percutaneous coronary intervention.

**Supplementary Table S7**

**Sensitivity analysis - Multivariable cox analysis for recurrent ICD shock, adjusted for manufacturer**

|  | Recurrent any shock | P for interaction | Recurrent appropriate shock | P for interaction | Recurrent inappropriate shock | P for interaction |
| --- | --- | --- | --- | --- | --- | --- |
| Age per 10-year increment | 0.98 [0.84;1.13] 0.768 | 0.5483 | 0.98 [0.84;1.14] 0.803 | 0.571 | 1.15 [0.65;2.05] 0.622 | 0.799 |
| Female sex  (Ref: Male) | 1.15 [0.70;1.91] 0.574 | 0.0370* | 1.16 [0.69;1.94] 0.579 | 0.0431* | 0.67 [0.08;5.97] 0.722 | 0.999 |
| BMI- obese  (Ref: normal) | 1.36 [0.86;2.15] 0.183 | 0.421 | 1.23 [0.77;1.95] 0.381 | 0.389 | 1.05 [0.17;6.66] 0.956 | 0.359 |
| BMI- overweight (Ref: normal) | 1.07 [0.70;1.65] 0.7501 | 0.421 | 0.97 [0.63;1.50] 0.884 | 0.389 | 0.97 [0.19;4.95] 0.971 | 0.359 |
| BMI-underweight (Ref: normal) | 1.15 [0.15;8.68] 0.892 | 0.421 | 1.17 [0.15;8.86] 0.878 | 0.389 | NA | 0.359 |
| Reduced LVEF  (Ref: LVEF >35) | 1.13 [0.77;1.66] 0.525 | 0.3376 | 1.15 [0.78;1.70] 0.492 | 0.3636 | 0.89 [0.16;4.86] 0.897 | 0.999 |
| Device (ref: ICD) | 0.59 [0.33;1.07] 0.082 | 0.9466 | 0.61 [0.33;1.13] 0.114 | 0.6920 | 1.04 [0.17;6.51] 0.963 | 0.999 |
| Revascularization  (ref: no PCI/CABG) | 1.46 [1.00;2.14] 0.0510 | 0.0133* | 1.59 [1.07;2.34] 0.0204* | 0.0114* | 0.59 [0.13;2.69] 0.492 | 0.262 |

Multivariable cox proportional hazard regression models for time from first to second of each outcome, for primary and secondary prevention together. P for interaction with primary and secondary prevention.

Results are given as HR [95% CI], P, and each model is adjusted for covariates as indicated by the given estimates, as well as manufacturer.

Abbreviations: BMI, body mass index; BMI category: underweight (BMI <18.5), overweight (BMI 25-29.9), obese (BMI ≥ 30.0), normal weight (BMI 18.5-24.9); CABG, coronary artery bypass graft; CRT-D, Implantable Cardioverter Defibrillator with cardiac resynchronization therapy; ICD, Implantable Cardioverter Defibrillator; LVEF, left ventricular ejection fraction (Reduced LVEF was defined as LVEF ≤35%); PCI, percutaneous coronary intervention.

**Supplementary Figure S1a**

**Event rates per 100 person-years for incident shocks, divided by ICD manufacturer**


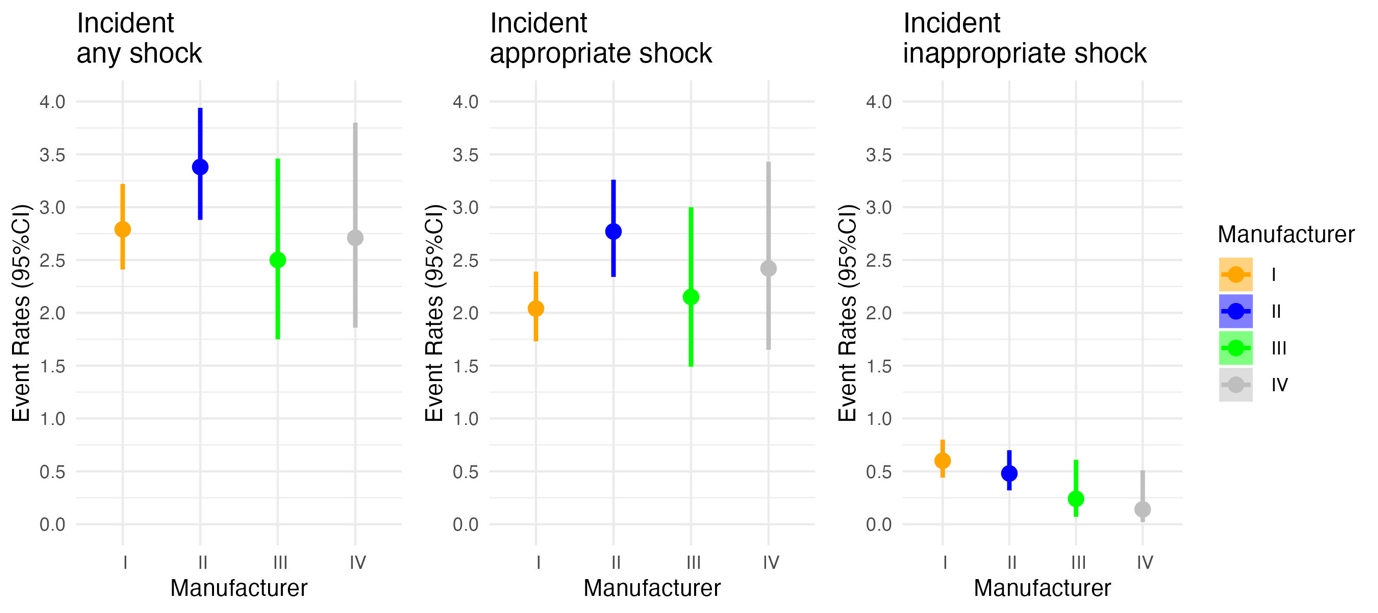


**Figure legend:** Event rates per 100 person-years for incident shocks (95% CI), stratified by manufacturer. Event rates per shock type did not statistically differ between manufacturers (reference: manufacturer I), except for manufacturer II versus I for incident appropriate shock (HR 1.36 [1.06;1.74] p=0.0149^*^)

^*^Multivariable Cox model adjusted for age, sex, BMI, LVEF, implant type, previous revascularization, and manufacturer.

Abbreviations: BMI, body mass index; CI, confidence interval, LVEF, left ventricular ejection fraction, HR, hazard ratio

**Supplementary Figure S1b**

**Event rates per 100 person-years for recurrent shocks, divided by ICD manufacturer**


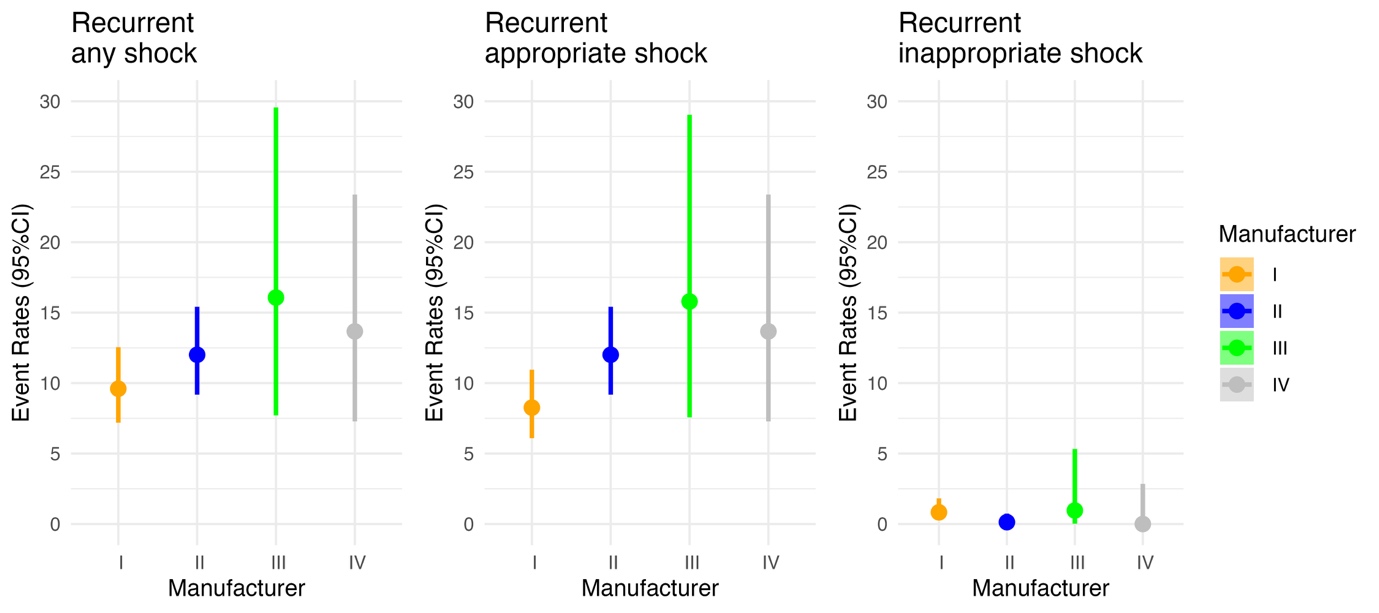


**Figure legend:** Event rates per 100 person-years for recurrent shocks (95% CI), divided by manufacturer. Event rates per shock type did not reach statistical difference between manufacturers (reference: manufacturer I). Multivariable Cox model adjusted for age, sex, BMI, LVEF, implant type, previous revascularization, and manufacturer.

Abbreviations: BMI, body mass index; CI, confidence interval, LVEF, left ventricular ejection fraction, HR, hazard ratio

**Supplementary Figure S2a**

**Event rates (95% CI) per 100 person-years for incident shocks between patients implanted before and after year 2015**


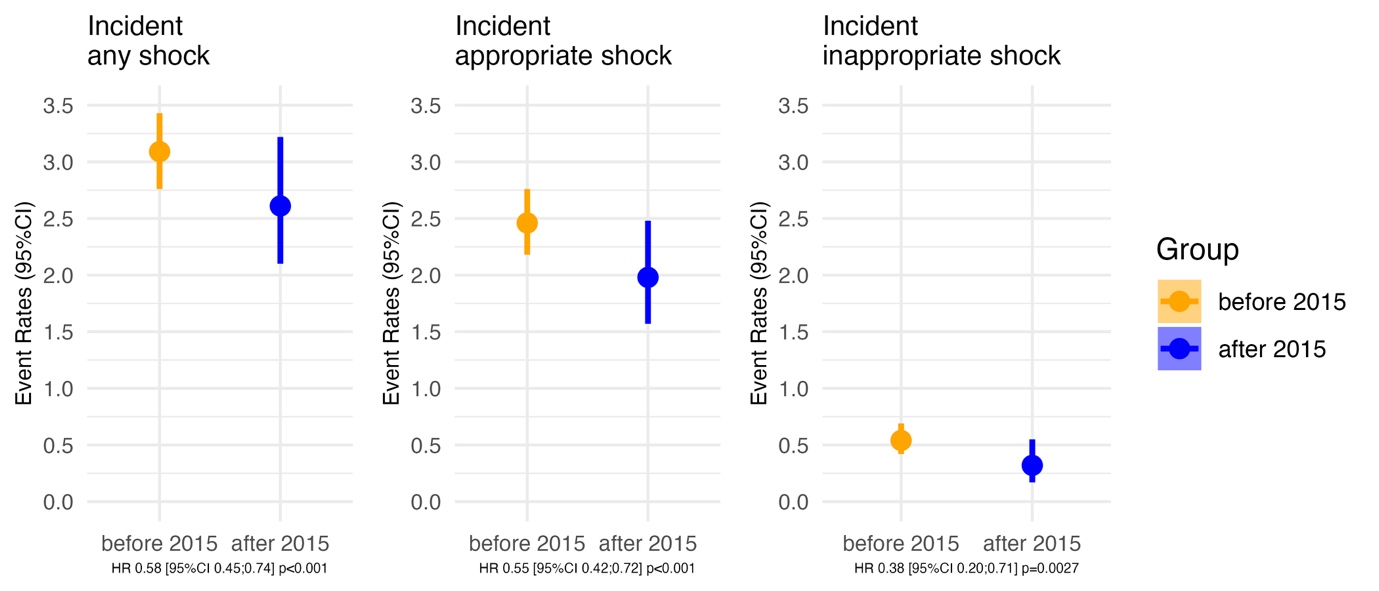


*All HR are adjusted for age, sex, BMI, LVEF, implant type and previous revascularization.

Abbreviations: BMI, body mass index; CI, confidence interval; LVEF, left ventricular ejection fraction, HR, hazard ratio

**Supplementary Figure S2b**

**Event rates (95% CI) per 100 person-years for recurrent shocks between patients implanted before and after year 2015**


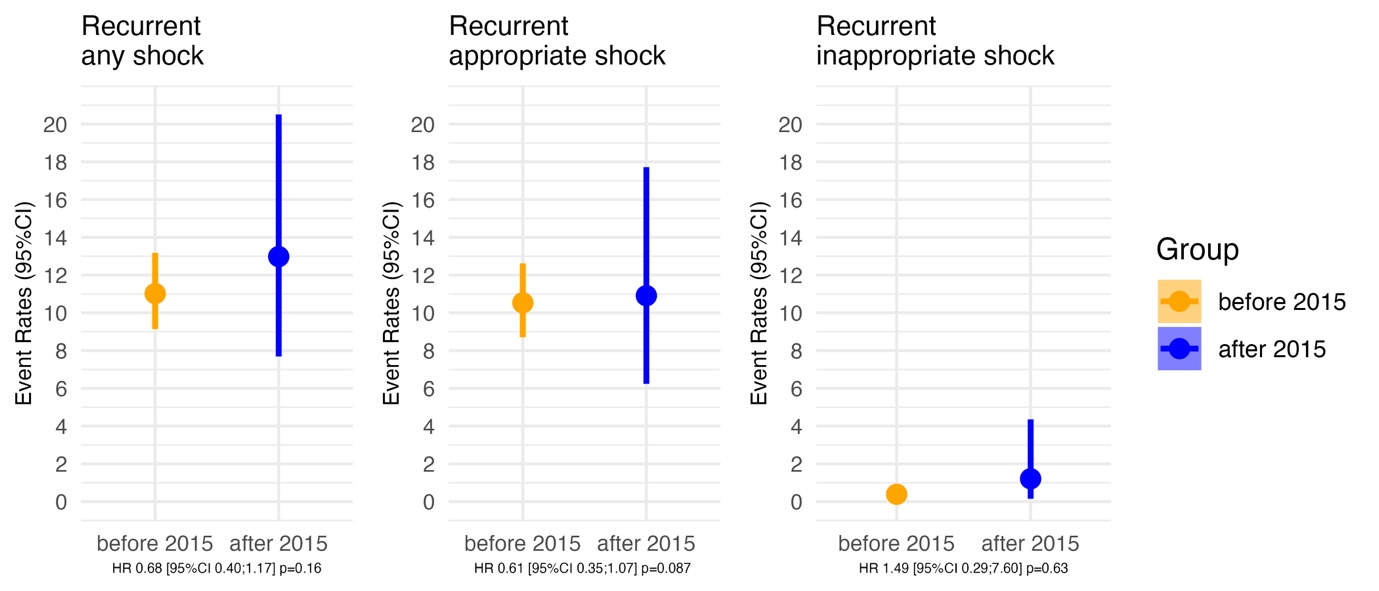


*All HR are adjusted for age, sex, BMI, LVEF, implant type and previous revascularization.

Abbreviations: BMI, body mass index; CI, confidence interval; LVEF, left ventricular ejection fraction, HR, hazard ratio
